# sEst: Accurate Sex-Estimation and Abnormality Detection in Methylation Microarray Data

**DOI:** 10.3390/ijms19103172

**Published:** 2018-10-15

**Authors:** Chol-Hee Jung, Daniel J. Park, Peter Georgeson, Khalid Mahmood, Roger L. Milne, Melissa C. Southey, Bernard J. Pope

**Affiliations:** 1 Melbourne Bioinformatics, The University of Melbourne, Parkville, VIC 3010, Australia; djp@unimelb.edu.au (D.J.P.); peter.georgeson@unimelb.edu.au (P.G.); khalid.mahmood@unimelb.edu.au (K.M.); bjpope@unimelb.edu.au (B.J.P.); 2 Department of Clinical Pathology, The University of Melbourne, Parkville, VIC 3010, Australia; 3 Cancer Epidemiology & Intelligence Division, Cancer Council Victoria, Melbourne, VIC 3004, Australia; Roger.Milne@cancervic.org.au (R.L.M.); msouthey@unimelb.edu.au (M.C.S.); 4 Centre for Epidemiology and Biostatistics, Melbourne School of Population and Global Health, The University of Melbourne, Parkville, VIC, 3010, Australia; 5 Precision Medicine, School of Clinical Sciences at Monash Health, Monash University, Clayton, VIC 3168, Australia; 6 Genetic Epidemiology Laboratory, The University of Melbourne, Parkville, VIC 3010, Australia

**Keywords:** DNA methylation, sex information, sex-chromosome abnormalities, epigenetics

## Abstract

DNA methylation influences predisposition, development and prognosis for many diseases, including cancer. However, it is not uncommon to encounter samples with incorrect sex labelling or atypical sex chromosome arrangement. Sex is one of the strongest influencers of the genomic distribution of DNA methylation and, therefore, correct assignment of sex and filtering of abnormal samples are essential for the quality control of study data. Differences in sex chromosome copy numbers between sexes and X-chromosome inactivation in females result in distinctive sex-specific patterns in the distribution of DNA methylation levels. In this study, we present a software tool, sEst, which incorporates clustering analysis to infer sex and to detect sex-chromosome abnormalities from DNA methylation microarray data. Testing with two publicly available datasets demonstrated that sEst not only correctly inferred the sex of the test samples, but also identified mislabelled samples and samples with potential sex-chromosome abnormalities, such as Klinefelter syndrome and Turner syndrome, the latter being a feature not offered by existing methods. Considering that sex and the sex-chromosome abnormalities can have large effects on many phenotypes, including diseases, our method can make a significant contribution to DNA methylation studies that are based on microarray platforms.

## 1. Introduction

DNA methylation is a major mechanism in the regulation of gene expression, and, consequently, plays an important role in disease susceptibility and progression [1,2]. The current pre-eminent DNA methylation microarray systems are Illumina’s Infinium HumanMethylation450 BeadChip (HM450k) and Infinium MethylationEPIC BeadChip (EPIC), which enable genome-wide analysis at relatively low cost, measuring the DNA methylation level of approximately 480,000 (HM450k) or 850,000 (EPIC) sites across the genome via site-specific oligonucleotide probes. Each site is associated with three values: a methylated signal; an unmethylated signal; and a detection *p*-value. The methylated and unmethylated signals are used to calculate methylation levels, represented as beta-values ranging from 0 (fully unmethylated status) to 1 (fully methylated status). The detection *p*-values indicate the quality of the signals and are determined by comparing the strength of signal to background noise.

Quality control is an important component of any screening approach, including the validation of specimen identity. Sex is one of the strongest influencers of the genomic distribution of DNA methylation and sample traits across differential effect studies and has a particular influence on predisposition, development, and prognosis for many diseases, including cancers [3,4]. However, many publicly available methylation datasets contain erroneous sex labels [5]. Qian and colleagues described a method for independently inferring the sex of screening specimens from single nucleotide polymorphism (SNP)-based microarray data [6]. The R-packages minfi [7] and RnBeads [8] are two of the most commonly used R packages for the analysis of HM450k data, providing the ‘getSex’ and ‘rnb.execute.gender.prediction’ functions, respectively. The ‘getSex’ function of minfi estimates the copy number of sex chromosomes from the total signal intensities (the sum of methylated and unmethylated signals) for the sex chromosomes and predicts sex as either male or female [7], expecting one X chromosome (chrX) and one Y chromosome (chrY) for typical males and two chrX and no chrY for typical females. Similarly, the ‘rnb.execute.gender.prediction’ function of RnBeads uses the increase in total signal intensities on sex-chromosomes compared to autosomes to infer sex [8]. However, the binary classification of sex by minfi and RnBeads is inappropriate in cases of sex-chromosome abnormalities, such as Klinefelter syndrome (males with two or more chrX) or Turner syndrome (females with only one chrX). Previous studies have shown that Klinefelter syndrome is the most common sex-chromosome disorder, affecting approximately 1 in 600 men, and that Turner syndrome occurs in approximately 1 in every 2000 live female births [9,10]. Klinefelter syndrome is an underdiagnosed condition [9], implying that it is not uncommon to inadvertently include Klinefelter syndrome individuals in studies featuring several hundred or more male samples. These abnormal sex-chromosome states should be properly accounted for in the analyses, since they confer increased risk for various diseases [11,12,13,14].

The difference in sex chromosome copy numbers between sexes and X chromosome inactivation (XCI) in females result in distinctive beta-value distributions between sexes regardless of tissue [15]. Additionally, the absence of chrY in females should have an apparent effect on the detection *p*-value distribution. In this study, we developed a software tool as an open source R package, sEst, that infers sex and sex chromosomal abnormalities from the distribution pattern of beta-values and detection *p*-values on the sex chromosomes.

## 2. Results

### 2.1. Clustering of Samples and Selection of Reference Samples

**Figure 1:**
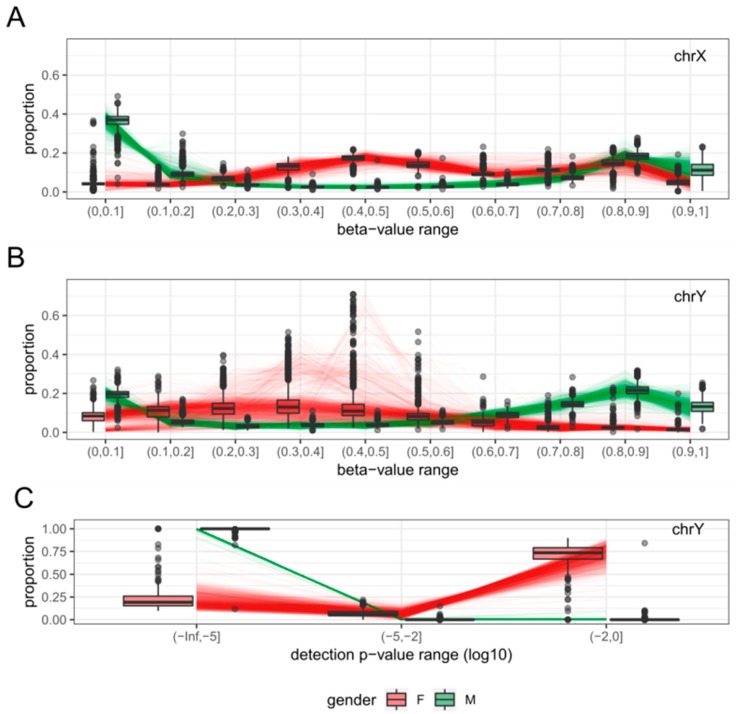
Distinct distributions of methylation levels and confidence measures at the sex chromosomes. Distribution profiles were generated from 1000 male and 1000 female samples randomly selected from 12 GEO datasets (See Materials and Methods for details). Each line represents the distribution profiles of male (green line) or female (red line), but the color was blurred in order to enhance the visibility of boxplot. (**A**) While the beta-value distribution on chrX of male samples is similar to the overall beta-value distribution for the whole genome (not shown), that of female samples is vastly different. (**B**) The beta-value distribution for male chrY is also similar to the genome-wide beta-value distribution, but its female counterpart is difficult to characterise because probes targeting chrY cannot yield credible signals from female samples. (**C**) The vast majority of chrY probes exhibit very low detection *p*-values for male samples, but the opposite is true for female samples.

**Figure 2:**
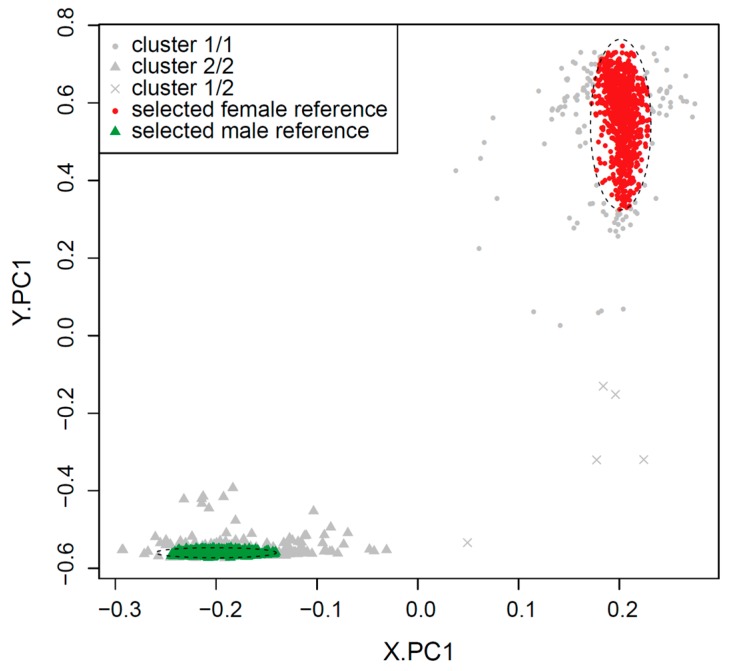
Selection of male and female reference samples. Randomly selected 2000 samples (1000 male and 1000 female) were plotted using the first principal component from PCA.X and PCA.Y (PCA.X on chrX and PCA on chrY, respectively), and those that were close to the centre of clusters were retained as male and female reference samples. Final reference samples are shown in color within ellipses with dashed lines. X.PC1 is the first principal component of PCA.X; Y.PC1 is the first principal component of PCA.Y.

A total of 2000 samples (1000 male samples and 1000 female samples) were selected at random from the datasets after quality control to examine the distribution patterns of each sex. For each of these samples, the proportion of probes within 10 equally distributed beta-value intervals (0 to 1 with increments of 0.1) and 3 detection *p*-value intervals (less than 1 × 10^−5^, 1 × 10^−5^ to 1 × 10^−2^ and 1 × 10^−2^ to 1) were calculated for each sex chromosome. The beta-value (on chrX and chrY) and detection *p*-value (on chrY) proportions exhibited different distributions between males and females (Figure 1). The combined distribution profile of the beta-values and detection *p*-values of chrX and chrY were submitted separately to principal component analysis (PCA.X and PCA.Y respectively) followed by k-means clustering (k = 2). When the clustering results from PCA.X and PCA.Y were combined, the vast majority of female-labelled samples (992 of 1000) were in cluster ‘1’ by both PCA.X and PCA.Y (‘1/1’), and the vast majority of male-labelled samples (998 of 1000) were in cluster ‘2’ by both PCA.X and PCA.Y (‘2/2’) (Table 1). When plotted using the first principal component of PCA.X and PCA.Y, cluster ‘1/1’ samples were clearly separate from cluster ‘2/2’ samples (Figure 2). Based on their relative proximity to the cluster centres, 873 of the 992 samples in cluster ‘1/1’ and 868 of the 998 samples in cluster ‘2/2’ were retained as reference female and reference male samples, respectively (Figure 2). The beta-value proportions for the reference samples were pre-calculated for 100 intervals (0 < β ≤ 0.01, 0.01 < β ≤ 0.02, …, 0.99 < β ≤ 1), and the detection *p*-value proportions for the reference samples were pre-calculated for 18 intervals (*p* ≤ 1 × 10^−17^, 1 × 10^−17^ < *p* ≤ 1 × 10^−16^, …, 0.1 < *p* ≤ 1). These pre-calculated proportion tables were used to build a reference proportion table according to the user-provided intervals for beta-value and detection *p*-value.

### 2.2. Sex Estimation Method

The beta-value and detection *p*-value distributions of male and female reference samples were used to estimate the sex of test samples according to the following Algorithm 1.


**Algorithm 1: sEst**
1: Build a table of beta-value/detection *p*-value distributions for new samples:Default beta-value (β) intervals: 0 < β ≤ 0.1, 0.1 < β ≤ 0.2, …, 0.9 < β ≤ 1Default detection *p*-value (*p*) intervals: *p* ≤ 1 × 10^−5^, 1 × 10^−5^ < *p* ≤ 0.01, 0.01 < *p* ≤ 12: Combine the distribution tables for new samples with that representing reference samples.3: Run principal component analysis (PCA) on beta-value and detection *p*-value distributions for each sex chromosome (PCA.X: PCA on chrX; PCA.Y: PCA on chrY).4: Run k-means clustering (k = 2) on PCA.X and assign ‘f’ to the test samples that cluster with female reference samples, and ‘m’ to those that cluster with male reference samples.5: Run k-means clustering (k = 2) on PCA.Y and assign ‘f’ to the test samples that cluster with female reference samples, and ‘m’ to those that cluster with male reference samples.6: Predict sex:‘F’, if the assigned sex from PCA.X and PCA.Y is ‘f’;‘M’ if the assigned sex from PCA.X and PCA.Y is ‘m’;‘N’ if the two assigned sexes conflict.

### 2.3. Features

The main function of the sEst package is ‘estimateSex’, which applies the sex-estimation method to the input samples. The sEst package also provides two functions for visualization. The ‘plotSexEstimation’ function plots the first component of the PCA of the chrX profiles (X.PC1) against the first component of the chrY profiles (Y.PC1) with or without the reference samples, and ‘plotSexDistribution’ generates a line chart showing the beta-value distribution for chrX and chrY and detection *p*-value distribution for chrY. More details and example codes can be found on the website for this tool [16].

### 2.4. Sex-Prediction in the Test Samples

**Figure 3:**
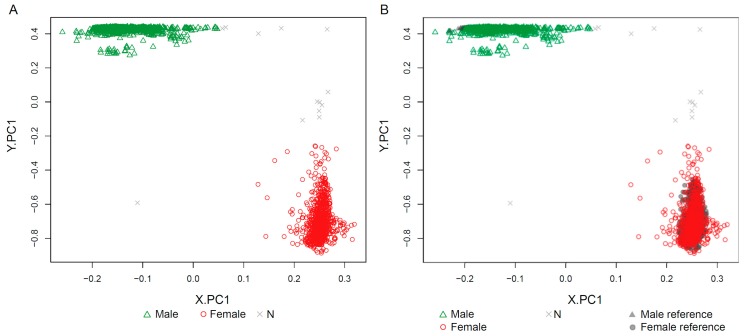
Plots from ‘plotSexEstimation’. This function plots the first component of PCA.X (X.PC1) against the first component of PCA.Y (Y.PC1). The vast majority of test samples were predicted as either M (2253) or F (1618), but the sex was undetermined for 20 samples, indicated as N. (**A**) The ‘include_reference’ parameter was set to FALSE to show only test samples. (**B**) The ‘include_reference’ parameter option was set to TRUE to show reference samples, but they were mostly hidden underneath test samples.

**Figure 4:**
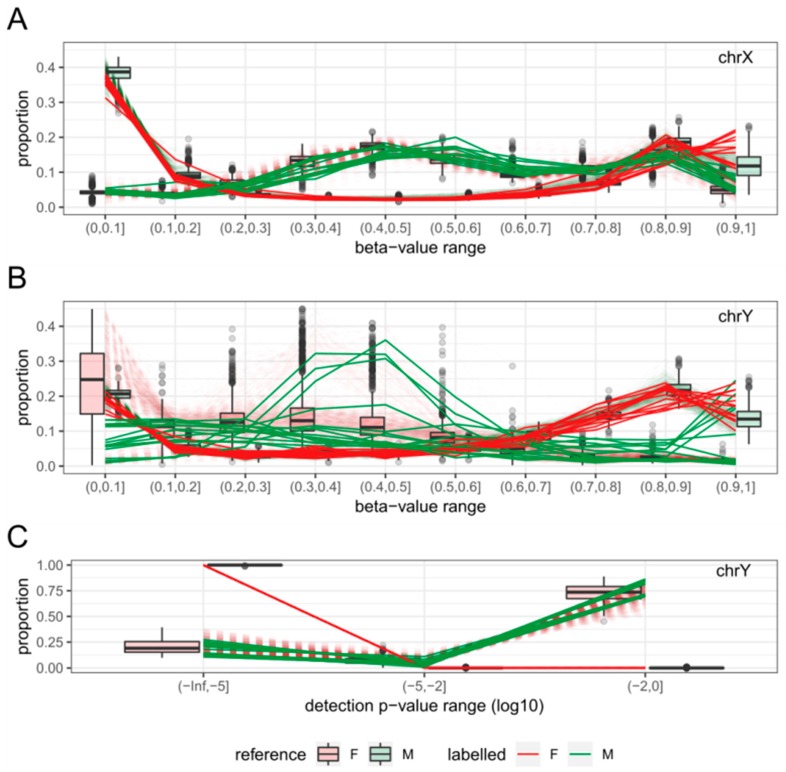
Beta-value/detection *p*-value distributions of discordantly predicted samples. The reference sample patterns are shown in boxplots on top of dashed lines in blurred red (F, Female) and green (M, Male), and those of discordant samples are shown in solid lines in the colours noted in the legend. Discordant males (predicted as F) follow the pattern of female reference samples, and discordant females (predicted as M) follow the pattern of male reference samples. (**A**) Distribution profiles of beta-values on chrX. (**B**) Distribution profiles of beta-values on chrY. (**C**) Distribution of detection *p*-values on chrY.

**Figure 5:**
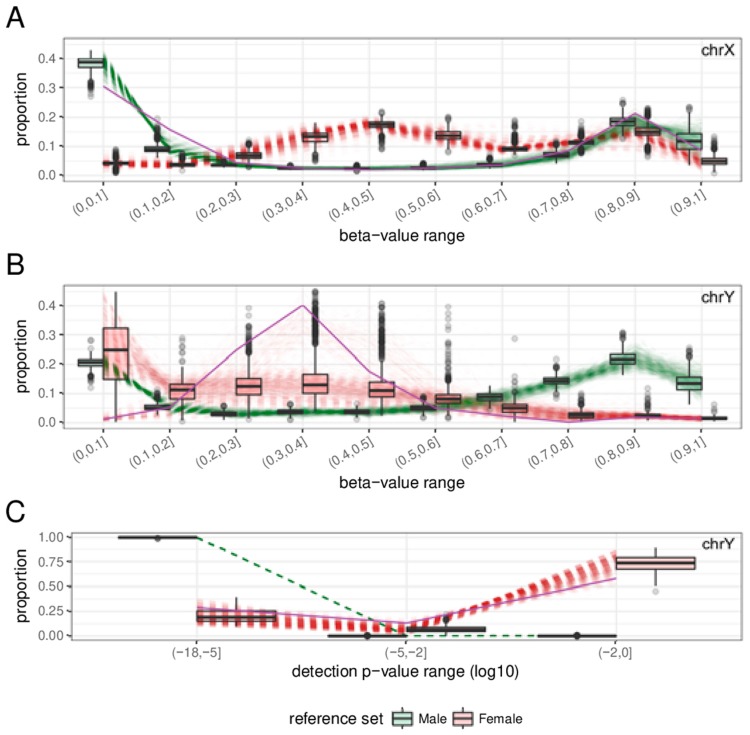
Beta-value/detection *p*-value distribution of the Turner syndrome sample. Sample GSM1572595, shown in purple, exhibits a male-like pattern for chrX (**A**) and a female-like pattern for chrY (**B**,**C**). Dashed green and red blurred lines show the profiles of reference samples.

The ‘estimateSex’ function was applied to 2788 test samples, which excluded the 2000 randomly selected samples from the 4788 quality-controlled samples from 12 GEO datasets (Figure 3). For the vast majority of samples with sex information, sEst predicted the sex as labelled (99.26% of male-labelled; 98.5% of female-labelled), and all 96 samples without sex information were estimated as either M (Male) or F (Female) (Table 2 and Figure S1). However, nine male-labelled samples and four female-labelled samples were predicted as the opposite sex (discordant) (Table 2), and 13 samples were predicted as ‘N’ (N-samples) (Table 2). The beta-value/detection *p*-value distributions of discordant samples clearly show the pattern of the predicted sex, suggesting that their sex was most likely mislabelled (Figure 4). The sex of seven discordant samples was re-estimated in a previous study, which used the methylation level of a few methylation sites known to be differentially methylated between sexes [5]. The re-estimated sex in the previous study was concordant with the prediction by sEst for five samples that were predicted as either M or F by sEst (Table S3). Eight of the 13 N-samples displayed clear female-like chrX and male-like chrY patterns, consistent with Klinefelter syndrome (male with more than one chrX). One N-sample (GSM1572595) displayed a male-like chrX pattern and a female-like chrY pattern, consistent with Turner syndrome (female with one chrX) (Figure 5 and Figure S2). Following the classification by sEst, we discovered that this sample had indeed been extracted from an individual with Turner syndrome in the corresponding study [17], thus validating our prediction. Four N-samples displayed slightly shifted beta-value distribution patterns but otherwise had very similar profiles to other male samples. These were re-estimated as male when wider beta-value intervals were applied (Figures S3 and S4).

### 2.5. Comparison with Existing Method

**Figure 6:**
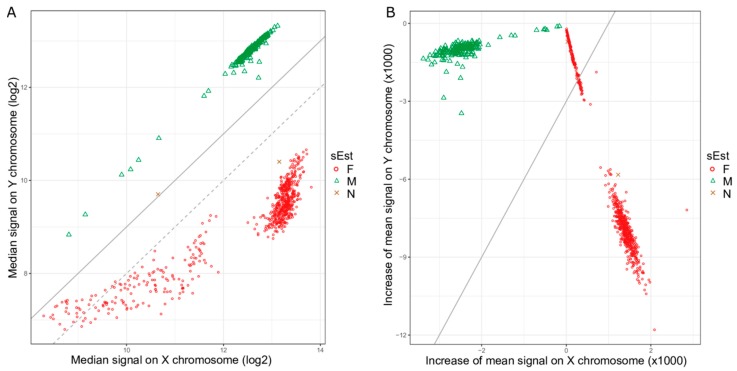
Signal intensity plots for dataset GSE51032. (**A**) The median signal intensity of chrX probes (xMed) against the median signal intensity of chrY probes (yMed) in log2-scale for each sample. The grey dotted line indicates the default cut-off of −2 for the difference between xMed and yMed (yMed−xMed) that the ‘getSex’ function of minfi package uses. A lot of red circles are above this line and are classified as Male by the ‘getSex’ function using the default parameters. Changing the cut-off to −1 (grey solid line) would reclassify the red circles above the grey dotted line as Female. (**B**) Increase in mean signal intensity on chrX and chrY compared to mean signal intensity on all autosomes. The grey solid line represents the cut-off line, which passed through a cluster of female samples, resulting in a large number of misclassified samples.

**Figure 7:**
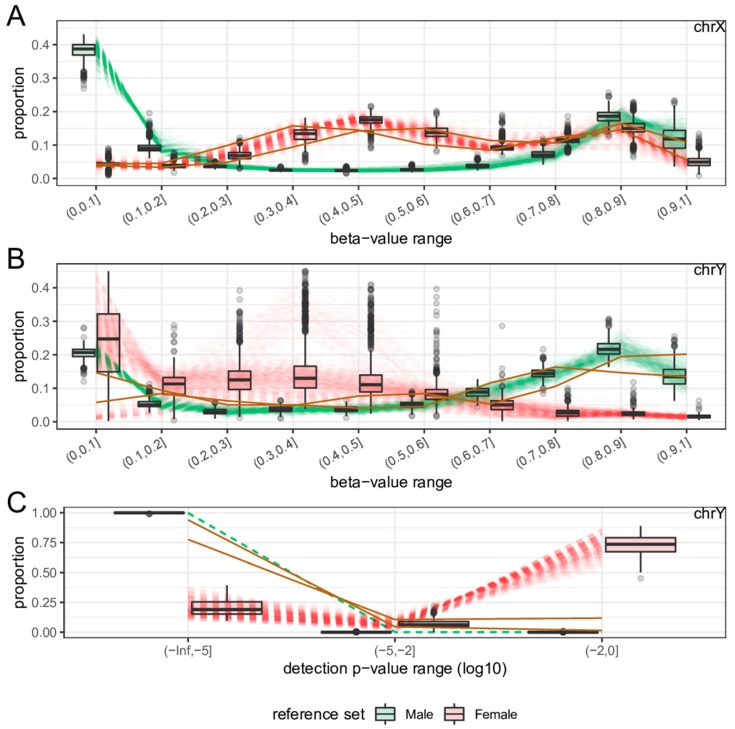
Beta-value/detection *p*-value distributions of two N-samples from dataset GSE51032. The patterns of N-predicted samples are shown in solid gold lines, and those of reference samples are shown in dashed lines and as boxplots. N-predicted samples show female-like chrX profiles (**A**) and male-like chrY profiles (**B**,**C**).

In addition to the 2788 test samples from 12 GEO datasets, raw data files (idat files) for 845 samples from GSE51032 were downloaded for the performance comparison of sEst with the ‘getSex’ function of the minfi package [7] and the ‘rnb.execute.gender.prediction’ function of the RnBeads package [8]. Their beta-values and detection *p*-values were extracted using the minfi package [7]. The quality-control procedure (see above) removed one sample due to a high percentage (>5%) of chrX probes with a detection *p*-value ≥ 0.01, leaving 844 samples for analysis. The sEst package predicted 655 samples as F, 187 samples as M, and two samples as N (Table 3). The two N-samples were labelled as female, and the female-predicted samples and male-predicted samples included five male-labelled samples and five female-labelled samples, respectively. Minfi’s ‘getSex’ function, using the default options, predicted 624 samples as F and 220 samples as M (Table 3), which was markedly different from both the sEst result and the prior sex-label. This discrepancy was caused by the smaller difference (in absolute value) between the median log2-signal intensity of chrX (xMed) and the median log2-signal intensity of chrY (yMed) for 32 female-predicted samples (Figure 6A). Changing the cut-off of ‘yMed minus xMed’ to ‘−1’ reclassified these 32 samples as Male, making the sex-predictions of ‘getSex’ consistent with those of sEst, except for the two N-samples (Table 3). The ‘rnb.execute.gender.prediction’ function of the RnBeads package also misclassified 128 samples (Figure 6B and Table 3), due to similar reasons as for the minif function. While the two N-samples were previously labelled as female, both ‘getSex’ and ‘rnb.execute.gender.prediction’ predicted one as F and the other as M (Table 3). However, the beta-value/detection *p*-value distribution profiles clearly showed female-like profiles for chrX and male-like profiles for chrY (Figure 7).

## 3. Discussion

Human error in labelling sample information occurs more frequently than one may assume, even for simple characteristics, such as sex [5]. As sex can have profound effects on study, mislabelling can result in false conclusions from the analysis of study data. In most cases, quality-control procedures preceding analysis should be able to identify and correct (or exclude) samples with mislabelled sex information. Illumina’s BeadChip-based platforms for DNA methylation profiling have been available for several years, and the ‘getSex’ method of sex-estimation has also been available for years within the package minfi [7]. Although ‘getSex’ successfully makes a correct sex-prediction in most cases, it is not effective at tagging samples with ambiguous patterns of beta-values and detection *p*-values on the sex chromosomes, as demonstrated in the present study. We have demonstrated that our method, sEst, significantly improves upon minfi by using typical beta-value and detection *p*-value distribution profiles for the sex-chromosomes as references to infer the sex of test samples and to detect samples with abnormal sex-chromosomes. Another R-package, RnBeads [8], also provides a function for sex-prediction, called ‘rnb.execute.gender.prediction’, which made an identical sex-prediction for the two N-samples of GSE51032 to the ‘getSex’ function of the minfi package. Given that the ‘rnb.execute.gender.prediction’ also uses signal intensity for sex-prediction, sEst holds the same advantages for sex-prediction over this package as it does over minfi. The reference profiles were compiled from a set of male and female samples that were randomly selected and refined from a large accumulation of HM450k data from public domains. These selected samples are from various tissues. However, this should have little to no effect to the reference profile, because the overall beta-value distribution is consistent across tissues as evidenced by the sex-chromosomes [15]. Although Illumina’s EPIC array, the successor of HM450k microarray, is becoming more popular for microarray-based methylation studies, we were unable to utilize EPIC array data for the development of this tool due to its scarcity in public data repositories. However, as the EPIC platform contains the vast majority of methylation sites targeted by the HM450k platform, sEst can be directly applied to EPIC array data. Combining the distribution profiles of test samples with those of reference samples for sex-prediction prevents issues that can arise from a dataset that is heavily biased towards either sex. The use of distributions of beta-values and detection *p*-values eliminates the need for raw data files (idat), which are needed by the minfi package but are often absent from public databases, and negates the requirement for normalisation as variation in methylation status at individual sites has minimal influence on the distribution profiles. The results of sEst consistently agree with sex predictions made by ‘getSex’ in cases where the methylation distribution profile is clearly either male-like or female-like. Furthermore, our method also tags samples that are too ambiguous for simple binary sex-prediction, alerting researchers to investigate further. This achieves a critical goal of quality control not well supported by prior methods. For example, one of the two N-samples in the GSE51032 dataset was located near the threshold line for sex-prediction by minfi and could possibly have attracted the attention of researchers using the dataset, but the other would hardly attract any attention for further examination as it was clustered with other female-predicted samples (Figure 6A), when in fact it shows male-like chrY profiles in the sEst result (Figure 7). While the lack of HM450k data from Klinefelter syndrome individuals in the public databases makes it hard to cross-check the N-samples in the GSE51032 dataset, the male-like chrY profiles and the female-like chrX profile suggest the existence of a single copy of chrY and two copies of chrX, whether as two whole chromosomes or as mosaicism, with one being heavily methylated. Two copies of chrX and one copy of chrY are the characteristics of Klinefelter syndrome. Klinefelter syndrome is the most common sex-chromosome disorder and yet is severely underdiagnosed [9], which implies that it would not be unlikely to have a number of Klinefelter syndrome samples in a study involving hundreds or more male samples.

The convenience and accuracy of sEst for sex-prediction and its ability to identify abnormal samples, which could be extracted from individuals with suspected Klinefelter syndrome or Turner syndrome, will make an important contribution to the accuracy of microarray-based DNA methylation data analyses, especially in contexts involving large sample numbers, including meta-analyses that combine datasets from public databases.

## 4. Materials and Methods

### Data Collection and Processing

Publicly available HM450k data for a total of 4801 samples, which were extracted from various normal tissues, were downloaded from 12 different sources in the Gene Expression Omnibus (GEO) [18,19] to derive beta-values and detection *p*-values (Table S1). These data include 3087 specimens labelled as male, 2460 labelled as female, and 99 samples without sex information. Raw beta-values were either directly downloaded or calculated from raw methylated and unmethylated signals using the method described by Du and colleagues [20]. Under the assumption that the proportion of probes with an unreliable detection *p*-value (≥0.01) should be consistent across all chromosomes, 13 samples that had a detection *p*-value ≥ 0.01 for more than 5% of chrX probes were excluded as a quality control measure, leaving 4788 samples for method development and testing.

## Figures and Tables

**Table 1:** Comparison of clustering results and labelled sex for 2000 randomly selected samples.

Labelled Sex	Cluster by PCA.X/Cluster by PCA.Y			
	1/1	1/2	2/1	2/2
Female	992	4	0	4
Male	1	1	0	998

**Table 2:** sEst-based sex estimation for test specimens.

Labelled	Predicted			Total
	F (%)	M (%)	N (%)	
F	787 (98.50)	4 (0.50)	8 (1.00)	799
M	9 (0.48)	1879 (99.26)	5 (0.26)	1893
(subtotal)	796 (29.57)	1883 (69.95)	13 (0.48)	2692
UNKNOWN	47 (48.96)	49 (51.04)	0 (0)	96
Total	843 (30.24)	1932 (69.30)	13 (0.47)	2788

Percentages were calculated relative to the total counts in the right-most column. N: N-samples which are the samples showing atypical patterns.

**Table 3:** Comparison of sex-estimation by sEst and existing methods.

sEst	Labelled		Minfi (Default)		Minfi (Cut-Off: −1)		RnBeads	
	F	M	F	M	F	M	F	M
F	650	5	623	32	655	0	527	128
M	5	182	0	187	0	187	0	187
N	2	0	1	1	1	1	1	1
Total	657	187	624	220	656	188	528	316

## Supplementary Materials

Supplementary materials can be found at http://www.mdpi.com/1422-0067/19/10/3172/s1.
