# Supplementary material for: sEst: Accurate Sex-Estimation and Abnormality Detection in Methylation Microarray Data
Source: Int J Mol Sci. 2018 Oct 15;19(10):3172. doi: 10.3390/ijms19103172 (PMC6213967; doi:10.3390/ijms19103172)
Supplement: Supplementary file 1 [file ijms-19-03172-s001.pdf]

International Journal of
Molecular Sciences

MDPI

sEst: Accurate sex-estimation and abnormality detection in methylation microarray data

Additional File 1

Supplementary Tables

Table S1. GEO datasets

GEO	Female	Male	UNKNOWN	Related publications
GSE36054	55	79	0	[1]
GSE36369	191	117	0	-
GSE39560	34	0	0	[2]
GSE41273	0	62	0	[3]
GSE48472	30	26	0	[4]
GSE50798	0	24	0	[5]
GSE52401	36	208	0	[6]
GSE53740	155	130	99	[7]
GSE55763	871	1840	0	[8]
GSE56105	301	313	0	[9]
GSE64495	76	37	0	[10]
GSE67393	54	63	0	[11]

Table S2. Comparison of clustering results and labelled sex for 2,000 randomly selected samples

Labelled sex	cluster by PCA.X / cluster by PCA.Y			
	1/1	1/2	2/1	2/2
Female	992	4	0	4
Male	1	1	0	998

Table S3. Discordant samples and N samples

GEO	GSM	gender	predicted	prediction in other study [12]
GSE36054	GSM880066	M	N	-
	GSM880118	M	N	-
GSE36369	GSM926560	F	M	-
	GSM926561	F	M	-
	GSM926564	M	F	-
	GSM926566	M	F	-
	GSM926567	M	F	-
	GSM926568	M	F	-
	GSM926569	M	F	-
GSE48472	GSM1179524	F	N	-
	GSM1179528	F	N	-
	GSM1179542	M	N	-
GSE53740	GSM1299660	M	F	F
	GSM1299719	M	F	F
	GSM1299768	M	F	-
	GSM1300551	F	M	M
GSE55763	GSM1343079	F	M	M
	GSM1343082	M	F	F
	GSM1344329	M	N	F
	GSM1345136	F	N	-
	GSM1345197	F	N	-
	GSM1345206	F	N	-
	GSM1345260	F	N	-
	GSM1345432	F	N	-
GSE64495	GSM1572595	F	N	F (Turner Syndrome)
GSE67393	GSM1649745	M	N	-

Supplementary Figures

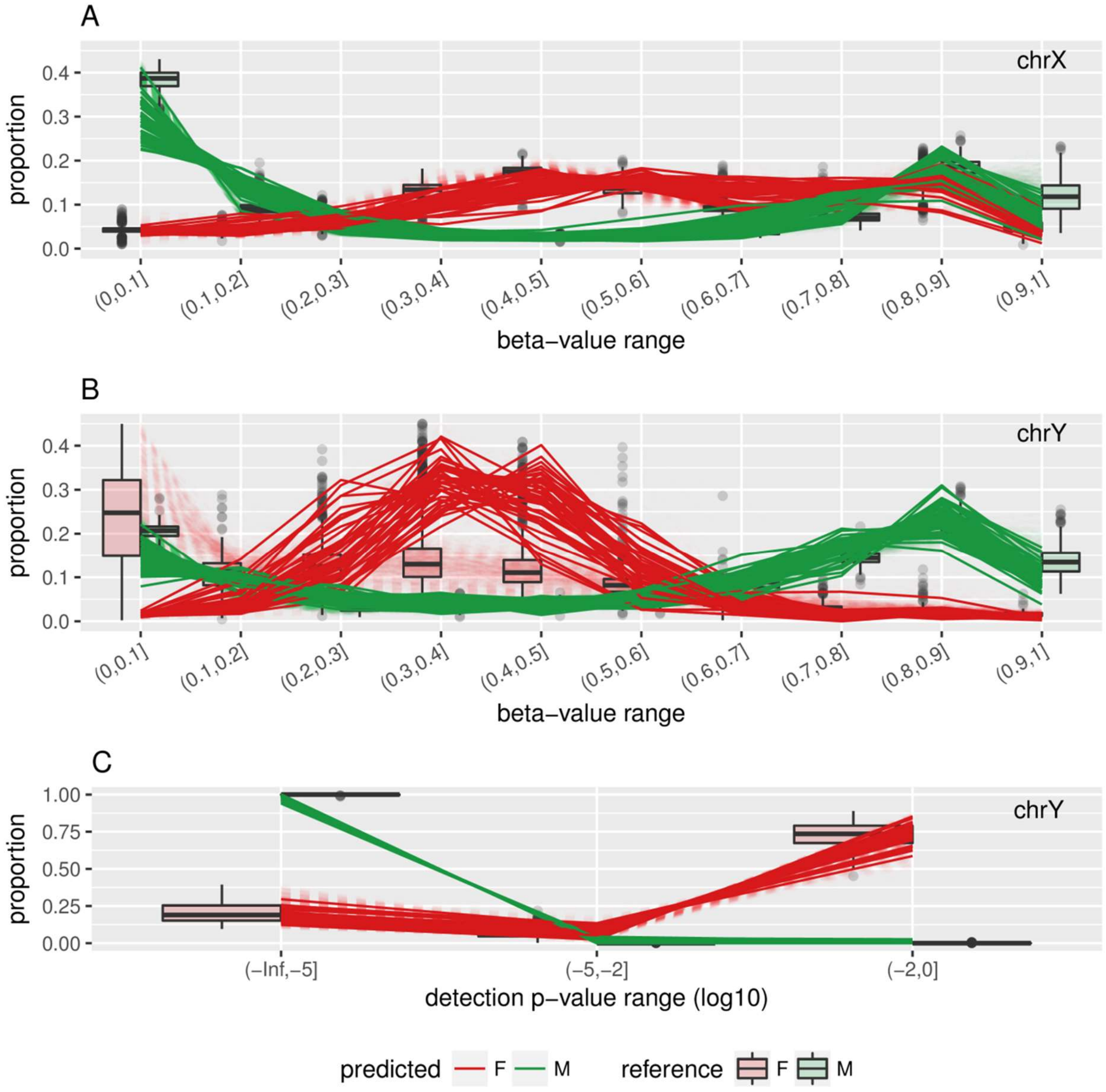

Figure S1. Beta-value/detection p-value distributions of test samples with UNKNOWN sex. All samples predicted as M show that the beta-distribution patterns of these samples are in good agreement with the patterns of the reference samples of the same sex.

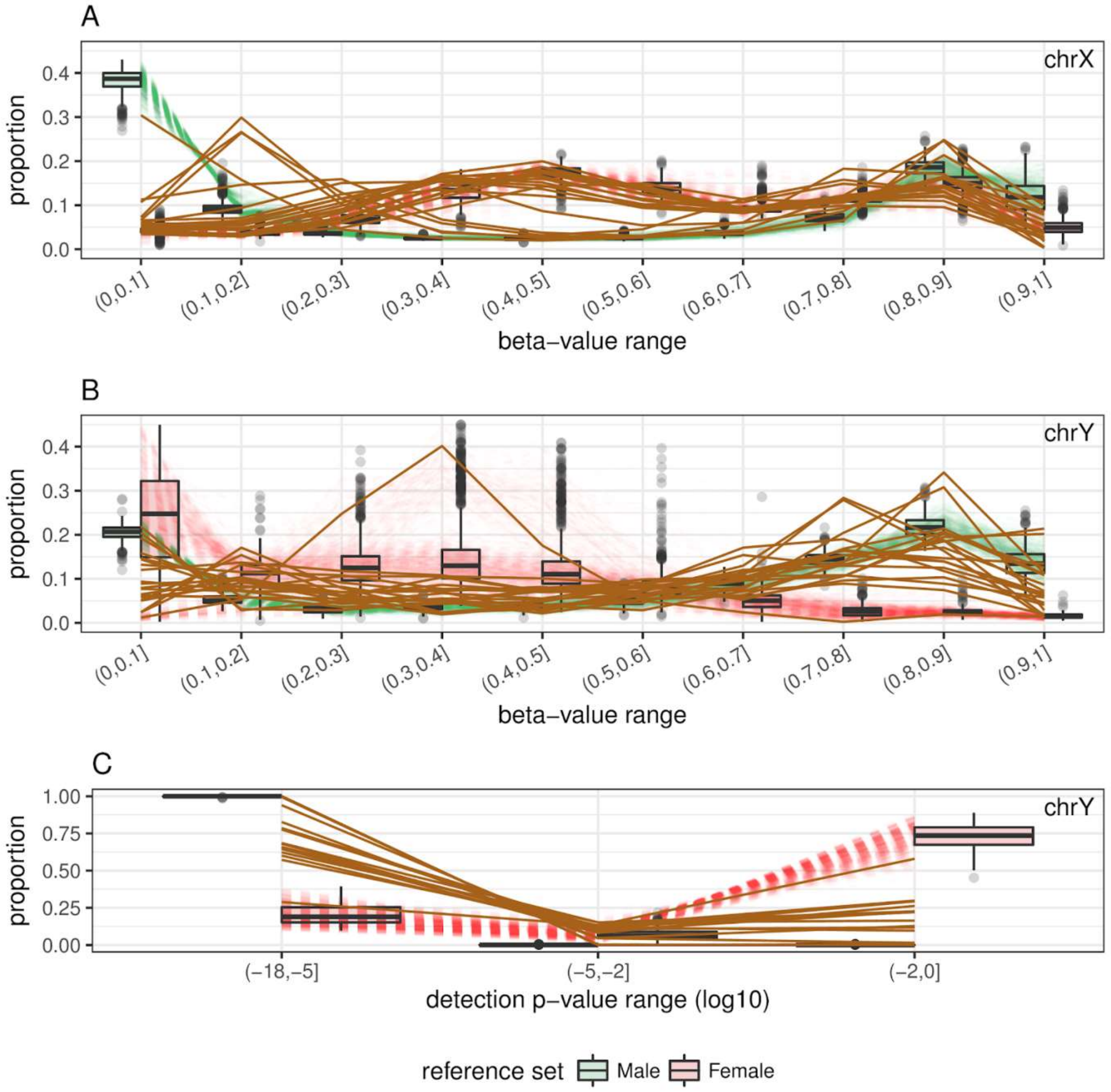

Figure S2. Beta-value/detection p-value distributions of N-samples shown in brown. While the majority have female-like chrX pattern (A), all N-samples except for one have male-like chrY (B, C). This plot was generated by 'plotSexDistribution' function.

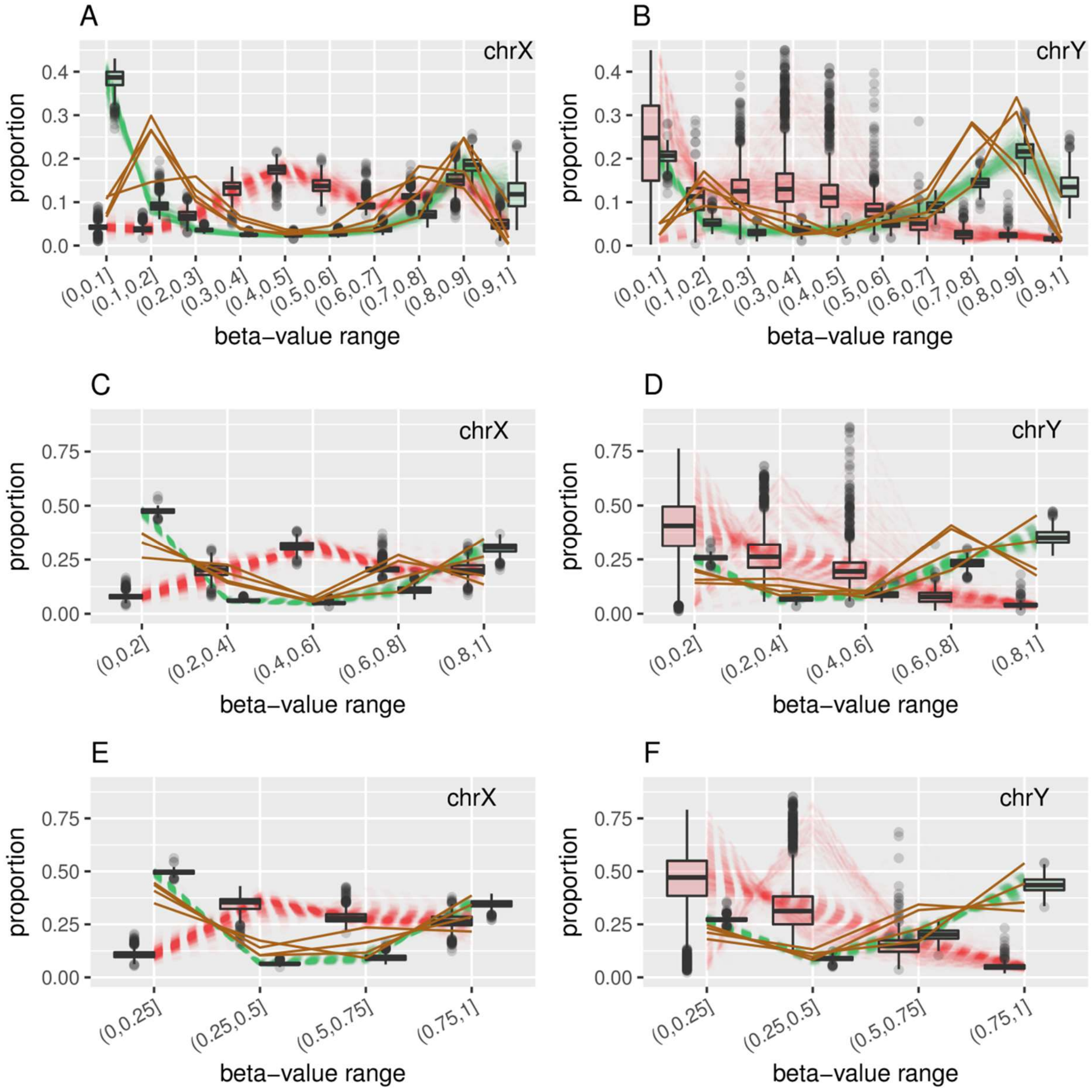

Figure S3. Beta-value distributions of N-samples that were re-estimated as M. Although appearing similar to male-like patterns, beta-value patterns of both chrX and chrY for four N-samples (dark yellow) were slightly shifted inwards (A,B). However, this variation was mitigated when larger beta-value interval ranges were used for gender estimation (C-F), resulting in re-estimation of these four N samples to M.

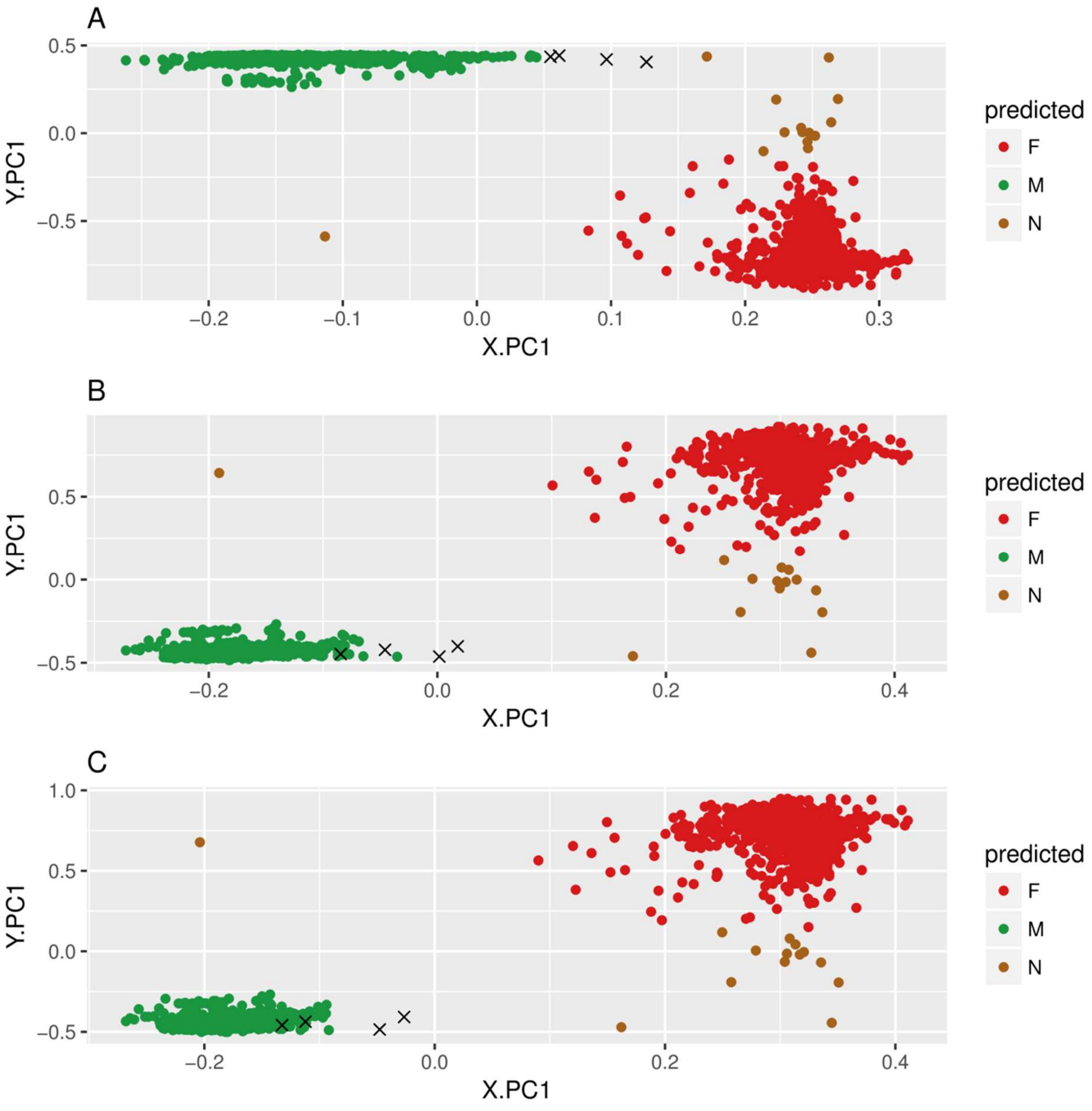

Figure S4. Plots of the first principal components in sex-estimation results with different beta-value intervals. The number of beta-value intervals is 10 (0 to 1 with the increment of 0.1) (A), 5 (0 to 1 with the increment of 0.2) (B) and 4 (0 to 1 with the increment of 0.25) (C). While the relative position of N-samples to the position of the male cluster (green dots) is almost stationary in all plots, 4 N-samples (shown in X) that were re-estimated as M with larger beta-value intervals moved closer to the male cluster as the beta-value interval increased.

References

1. Alisch, R. S.; Barwick, B. G.; Chopra, P.; Myrick, L. K.; Satten, G. A.; Conneely, K. N.; Warren, S. T. Age-associated DNA methylation in pediatric populations. Genome Res. 2012, 22, 623–632.
2. Souren, N. Y. P.; Lutsik, P.; Gasparoni, G.; Tierling, S.; Gries, J.; Riemenschneider, M.; Fryns, J.-P.; Derom, C.; Zeegers, M. P.; Walter, J. Adult monozygotic twins discordant for intra-uterine growth have indistinguishable genome-wide DNA methylation profiles. Genome Biol. 2013, 14, R44.

3. Alisch, R. S.; Wang, T.; Chopra, P.; Visootsak, J.; Conneely, K. N.; Warren, S. T. Genome-wide analysis validates aberrant methylation in fragile X syndrome is specific to the FMR1 locus. BMC Med. Genet. 2013, 14, 18.
4. Slieker, R. C.; Bos, S. D.; Goeman, J. J.; Bovée, J. V.; Talens, R. P.; van der Breggen, R.; Suchiman, H. E. D.; Lameijer, E.-W.; Putter, H.; van den Akker, E. B.; Zhang, Y.; Jukema, J. W.; Slagboom, P. E.; Meulenbelt, I.; Heijmans, B. T. Identification and systematic annotation of tissue-specific differentially methylated regions using the Illumina 450k array. Epigenetics Chromatin 2013, 6, 26.
5. Kozlenkov, A.; Roussos, P.; Timashpolsky, A.; Barbu, M.; Rudchenko, S.; Bibikova, M.; Klotzle, B.; Byne, W.; Lyddon, R.; Di Narzo, A. F.; Hurd, Y. L.; Koonin, E. V.; Dracheva, S. Differences in DNA methylation between human neuronal and glial cells are concentrated in enhancers and non-CpG sites. Nucleic Acids Res. 2014, 42, 109–127.
6. Shi, J.; Marconett, C. N.; Duan, J.; Hyland, P. L.; Li, P.; Wang, Z.; Wheeler, W.; Zhou, B.; Campan, M.; Lee, D. S.; Huang, J.; Zhou, W.; Triche, T.; Amundadottir, L.; Warner, A.; Hutchinson, A.; Chen, P.-H.; Chung, B. S. I.; Pesatori, A. C.; Consonni, D.; Bertazzi, P. A.; Bergen, A. W.; Freedman, M.; Siegmund, K. D.; Berman, B. P.; Borok, Z.; Chatterjee, N.; Tucker, M. A.; Caporaso, N. E.; Chanock, S. J.; Laird-Offringa, I. A.; Landi, M. T. Characterizing the genetic basis of methylome diversity in histologically normal human lung tissue. Nat. Commun. 2014, 5, 3365.
7. Li, Y.; Chen, J. A.; Sears, R. L.; Gao, F.; Klein, E. D.; Karydas, A.; Geschwind, M. D.; Rosen, H. J.; Boxer, A. L.; Guo, W.; Pellegrini, M.; Horvath, S.; Miller, B. L.; Geschwind, D. H.; Coppola, G. An epigenetic signature in peripheral blood associated with the haplotype on 17q21.31, a risk factor for neurodegenerative tauopathy. PLoS Genet. 2014, 10, e1004211.
8. Lehne, B.; Drong, A. W.; Loh, M.; Zhang, W.; Scott, W. R.; Tan, S.-T.; Afzal, U.; Scott, J.; Jarvelin, M.-R.; Elliott, P.; McCarthy, M. I.; Kooner, J. S.; Chambers, J. C. A coherent approach for analysis of the Illumina HumanMethylation450 BeadChip improves data quality and performance in epigenome-wide association studies. Genome Biol. 2015, 16, 37.
9. McRae, A. F.; Powell, J. E.; Henders, A. K.; Bowdler, L.; Hemani, G.; Shah, S.; Painter, J. N.; Martin, N. G.; Visscher, P. M.; Montgomery, G. W. Contribution of genetic variation to transgenerational inheritance of DNA methylation. Genome Biol. 2014, 15, R73.
10. Walker, R. F.; Liu, J. S.; Peters, B. A.; Ritz, B. R.; Wu, T.; Ophoff, R. A.; Horvath, S. Epigenetic age analysis of children who seem to evade aging. Aging 2015, 7, 334–339.
11. Inoshita, M.; Numata, S.; Tajima, A.; Kinoshita, M.; Umehara, H.; Yamamori, H.; Hashimoto, R.; Imoto, I.; Ohmori, T. Sex differences of leukocytes DNA methylation adjusted for estimated cellular proportions. Biol. Sex Differ. 2015, 6, 11.
12. Kim, J. H.; Park, J.-L.; Kim, S.-Y. Non-negligible Occurrence of Errors in Gender Description in Public Data Sets. Genomics Inform. 2016, 14, 34–40.
